# Combining an Intensive Green Roof with Seismic Retrofitting of Typical Reinforced Concrete Buildings in Israel

**DOI:** 10.3390/ma15030889

**Published:** 2022-01-24

**Authors:** Svetlana Pushkar, Ido Halperin, Yuri Ribakov

**Affiliations:** 1 Department of Civil Engineering, Ariel University, Ariel 40700, Israel; svetlanap@ariel.ac.il (S.P.); ribakov@ariel.ac.il (Y.R.); 2 Faculty of Architecture and Town Planning, Technion Israel Institute of Technology, Haifa 3200003, Israel

**Keywords:** retrofitted buildings, intensive green roofs, waste-based materials, seismic design, life-cycle analysis, ANOVA

## Abstract

This study suggests an intensive green roof as part of a sustainable and hazard-resistant conceptual design for the retrofitting of old buildings in Israel. The roof is suggested to be built with waste-based materials. A five-story reinforced concrete residential building was retrofitted with: Case 1: concrete wall strengthening (CWS)-conventional concrete + conventional green roof; Case 2: CWS-waste-included concrete + waste-based green roof; Case 3: seismic isolation columns (SIC)-conventional concrete + conventional green roof; and Case 4: SIC-waste-included concrete + waste-based green roof. Palekastro, Nuweiba, Tabas, and Erzincan ground motions were used for a structural dynamic time-history analysis of the retrofitted buildings. Life cycle assessments of cases 1–4 were performed using ReCiPe 2016 midpoint and endpoint evaluations. A two-stage analysis of variance (ANOVA) was used to analyze the ReCiPe endpoint results. According to the seismic results, Case 3 and Case 4 were much more preferable to Case 1 and Case 2, whereas according to the environmental evaluations, Case 4 was the most preferable to the other cases.

## 1. Introduction

Nowadays, the seismic retrofitting of old buildings toward their improved hazard-resistant design is an important issue in many countries. Israel uses a nationwide statutory plan known as Tama 38 for retrofitting buildings built during the 1970s [1]. According to Tama 38, building practitioners should add reinforced concrete (RC) stiffening elements to the retrofitted structure [2]. Except for this conventional method, there are certain modern retrofitting methods which are expected to supersede the older ones. Installing dampers that are based on the energy dissipation principle, such as high-damping rubber bearings that isolate the structure from ground motion [2,3,4], are examples of such contemporary approaches.

Recently, in addition to the improved seismic capacity they provide, the environmental performance of retrofitting methods has become a very pressing issue. Plainly, this is because they are based on concrete and steel, whose production might significantly harm the environment. For example, the production of 1 kg of Portland cement and 1 kg of steel can release 0.833 and 1.106 kg CO_2_-equivalent, respectively, thereby increasing the global warming potential (GWP) [5].

In this respect, Wei et al. [6] conducted a life cycle assessment (LCA) of CO_2_ emissions related to the conventional concrete retrofitting jacketing method. Vitiello et al. [7] studied LCAs of fiber-reinforced polymer (FRP)-based strengthening sheets, FRP-reinforced concrete (RC) jacketing, RC shear wall, rubber bearings, and friction isolators, applied to a reinforced building in Italy. They concluded that the rubber bearings and friction isolators were the most sustainable alternatives. Salgado et al. [8] studied RC column jacketing, RC shear walls and beam weakening, revealing that the shear wall strengthening was the unsustainable alternative. Ribakov et al. [2,9] analyzed concrete wall strengthening (CWS) and viscous dampers connected to steel chevron braces (VD&SB) and to concrete chevron braces (VD&CB), high-damping rubber bearings (HDRBs), and seismic isolation columns (SIC). It was reported that VD&SB, VD&CB, HDRBs, and SIC were more sustainable alternatives than CWS.

Welsh-Huggins and Liel [10] suggested incorporating green roofs as an additional element to hazard-resistant design toward further development in a sustainable seismic design. A typical office building in Los Angeles was designed with intensive and extensive green roofs (a new building) and extensive green roof (a retrofitted building). The average roof dead load for the intensive green roof was 1000 kg/m^2^ due to the deeper soil layer (up to 200 cm), whereas for the extensive green roof, it was 250 kg/m^2^ due to the shallower soil layer (up to 20 cm). Also, the average live load for the intensive green roof was higher than for the extensive green roof (500 and 100 kg/m^2^, respectively). Thus, Welsh-Huggins and Liel [10] concluded that in contrast to the extensive green roof, the building with intensive green roof manifested an improved seismic performance. However, these results were relevant only for a typical new building located in Los Angeles, which had a configuration specific to the city.

The aforementioned sustainable seismic design studies are country-specific because of the country-dependent nature of local building technologies, building configurations, and probable seismic events. Therefore, their findings cannot be generalized across all building configurations and countries [10]. In this respect, the present paper suggests a new concept of seismic as well as environmental solution–incorporation of an intensive green roof into the seismic design of retrofitted buildings in Israel. Such seismic design incorporating a green roof has not been environmentally evaluated yet for a retrofitted building. The substantial of the analysis, conducted here, is conceptual. It discusses a class of buildings, rather than a specific structure and intends to provide incipient results, which will underpin future studies.

## 2. Methods

### 2.1. Research Aims and Framework

This paper presents results of a conceptual study inspecting the inclusion of an intensive green roof into the retrofitting of typical, earthquake susceptible, existing old buildings in Israel. This class of buildings is represented here by a symmetric model of a five-story RC residential building, which accords construction approaches accepted in Israel during the 1970s and 1980s. As mentioned above, the main concern of this study is a new concept of seismic and environmental retrofitting. It includes a seismic resistance and environmental impact evaluation of the applied retrofitting. 

The following two seismic retrofitting alternatives were selected—CWS and seismic isolation columns (SIC)—and bring into account an addition of an intensive green roof to the structure. Replacing natural aggregates with recycled aggregates in the considered hazard-resistant design is also under consideration in this evaluation.

To sum up, the retrofitted design alternatives considered here are: (1) Case 1: CWS-conventional concrete + conventional intensive green roof; (2) Case 2: CWS-waste-included concrete + waste-based intensive green roof; (3) Case 3: SIC-conventional concrete + conventional intensive green roof; and (4) Case 4: SIC-waste-included concrete + waste-based intensive green roof. Table 1 provides an overview of these retrofitting-material combinations. CWS-conventional concrete, SIC-conventional concrete, and conventional intensive green roofs contains natural aggregates such as gravel, perlite, and sand, whereas CWS-waste-included concrete, SIC-waste-included concrete, and waste-based intensive green roofs contain recycled aggregates and waste materials such as concrete waste-based aggregates (CWAs), fly ash-based aggregates (FAAs), coal bottom ash (CBA), and fly ash (FA). The original, non-retrofitted structure, serves as the control case.

Palekastro (October 2021, Greece), Nuweiba (1995, Egypt), Tabas (1978, Iran) and Erzincan (1992, Turkey) ground motions were used for structural dynamic time-history analysis of the retrofitted buildings. Life cycle analysis (LCA) was conducted to evaluate the environmental damage related to the four retrofitted design alternatives. The LCA results of the retrofitted design alternatives were compared through a two-stage nested mixed ANOVA model.

### 2.2. CWS and SIC

During the 1970s and 1980s, many residential mid-rise RC buildings in Israel were constructed with open ground floor and slender columns. These buildings usually suffer from seismic vulnerability, do not comply with the modern seismic-design codes, and therefore require suitable engineering intervention.

**Figure 1:**
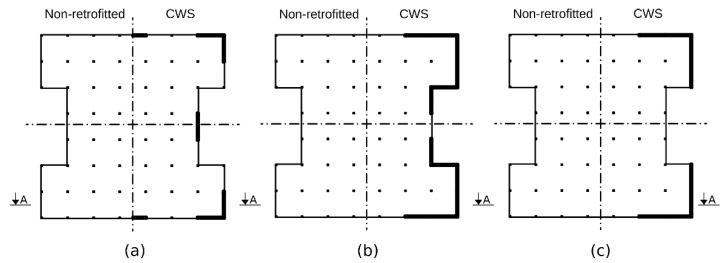
Floor plans of the analyzed typical building: on the right, concrete wall strengthening (Cases 1 and 2); on the left, the non-retrofitted structure (control case). (**a**) Ground floor (20 cm thick walls), (**b**) first floor (10 cm thick walls), and (**c**) floors two to four (10 cm thick walls).

The model, analyzed in this work, represents such a building. Its ground floor is open and, besides stairway walls, comprises only square-sectioned, 30/30 cm columns, made of C20 concrete. Each floor contains 60 columns. Their concrete Young’s Modulus is 23.8 GPa [11]. The floors’ heights are 2.6 m and the spans are 6 m in both directions. The floor’s area is 1548 m^2^. The overall mass of a typical story is 1904 ton and of the roof, 1333 ton. It is assumed that internal infill walls do not contribute to the structural system’s rigidity as a long time had passed since the structure’s construction, as well, low-quality construction and supervision place a question on their ability to operate as part of the lateral load bearing system. These assumptions lead to a structure whose mechanical properties can be described by a symmetric model. A schematic of this model is depicted in the left side of Figure 1a–c where the dashed-dotted lines represent the planes of symmetry. The right half of the non-retrofitted model is the left’s reflection. A planar dynamic model was chosen by virtue of the above symmetry. In accordance with common structural practice [12], the ceilings are assumed to be rigid and the mass is concentrated at the ceilings’ levels. All the above boils down to a dynamic shear model whose details are provided below, in Section 2.4. 

**Figure 2:**
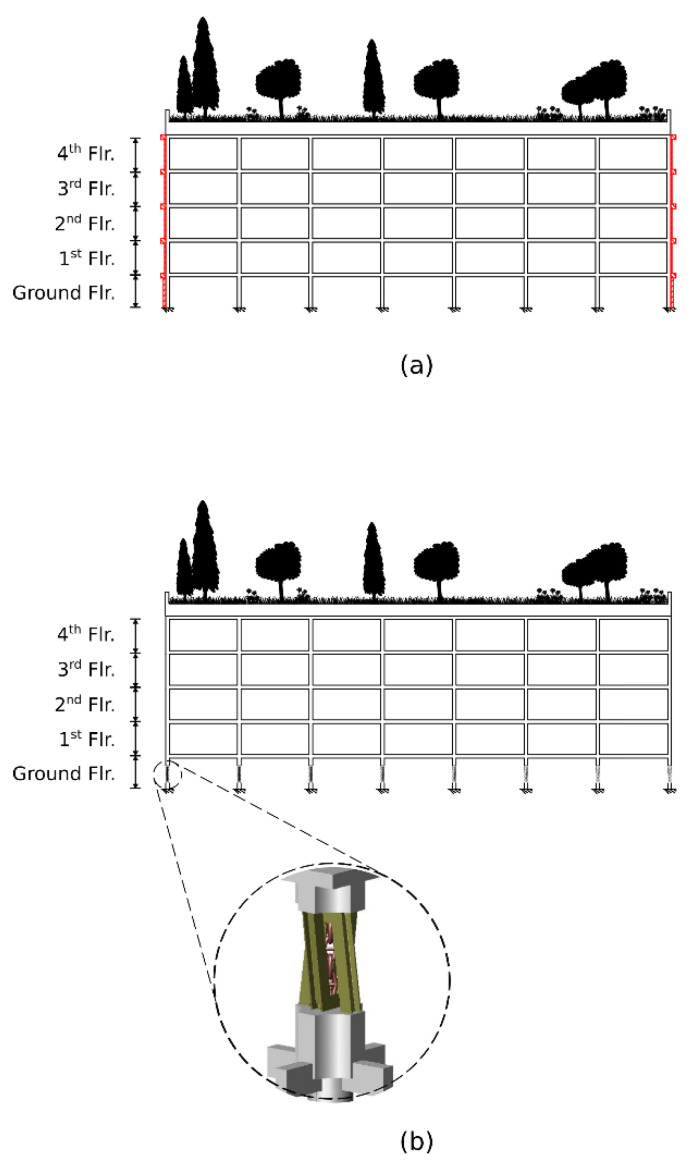
Vertical building section (A-A) of (**a**) concrete wall strengthening with deep green roof (Cases 1 and 2) and (**b**) SIC retrofitted structure with a deep green roof (Cases 3 and 4).

**Figure 3:**
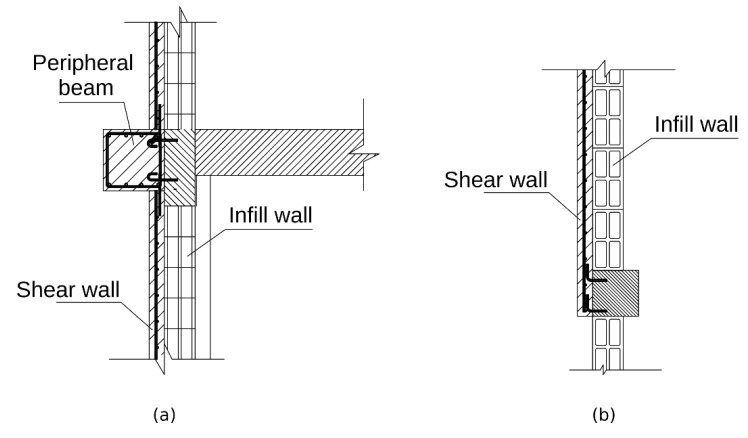
Concrete wall strengthening (Cases 1 and 2); vertical section of typical shear wall and peripheral beams (**a**); and horizontal section of typical shear wall-existing column interface (**b**).

The CWS method, which is rather classical and common, is based on strengthening the structure through improved strength elements, mainly concrete diaphragms. This is the retrofitting used in Cases 1 and 2. A vertical section of this configuration is depicted in Figure 2a. While the same live load, 490 kg_f_/m^2^, is applied to both cases, the dead load is different, due to the different compound of materials used in each one. In Case 1, the dead load is 2340 kg_f_/m^2^, whereas in Case 2, it is 1945 kg_f_/m^2^. However, the distribution of the strengthening RC diaphragms and the other retrofitting elements remains identical. It is illustrated by the right side of Figure 1. The left half of the CWS-retrofitted model is the right’s reflection. The retrofitting retains the structure’s symmetricity and accounts for two main practical issues, commonly evoked in such retrofitting. First, many structures slated for retrofitting are already occupied, thereby reducing the number of structural elements that are accessible for retrofitting operations. That is why in many retrofitting solutions, braces are implemented at the perimeter of the structure and in its internal staircase, as they are usually more accessible than other places. Another problem is that in many buildings, the ground floor is used for car parking. Hence, even though the elements in such a floor are more accessible, the need for parking places conflicts with the need to add stiffening walls, as it would block parking spots. In the suggested retrofitting, RC diaphragms are placed only at the outer walls, and no retrofitting elements are placed in the internal staircase. Such retrofitting is found sufficient for this case from a performance point of view. The number of RC diaphragms on the ground floor is reduced by half, compared to the first floor, for minimizing their impact on the parking places. However, in order to compensate for the reduced stiffness and strength, the thickness of the added diaphragms in this floor is doubled, and the perimeter beams in its ceiling level are modified accordingly. Shotcreting [13] of C30 concrete on existing walls is assumed as a method for forming these RC diaphragms. In total, 12 diaphragms of 20 cm thickness are embedded at the ground floor, 24 of 10 cm at the first floor, and 16 of 10 cm at each of the other floors. Additionally, in order to bond the reinforcing elements into a structural system, 32 beams are added at the perimeter of each ceiling. The beams’ cross-section is square, 40/40 cm in dimension, and are made of C30 concrete. Typical reinforcing elements are shown in Figure 3.

Seismic isolation by seismic isolation columns (SIC) [14] is used for increasing the seismic capacity in Cases 3 and 4. Their schematic is depicted in Figure 2b. These isolators were especially developed for retrofitting buildings with open ground floor, as in the control model. They are founded on the well-known concept of friction-pendulum [15]. Essentially, the idea is to transform each existing column into a seismic-isolation device by replacing its middle portion by two steel V-shaped frames, connected by a high-load bearing chain. The remaining parts of the column are jacketed by concrete to increase their load capacity. As in Cases 1 and 2, the retrofitting of Cases 3 and 4 is identical, except for a difference in the roofs’ dead loads. The roof dead load for Case 3 is 2340 kg_f_/m^2^ and for Case 4, it is 1945 kg_f_/m^2^.

A total of 288.2 m^3^ of concrete and 22.5 tons of steel are required by the CWS retrofitting, and 19.2 m^3^ of concrete and 22.3 tons of steel by the SIC retrofitting. The mix proportions for conventional concrete with a density of 2421 kg/m^3^ and compressive strength of 43.2 MPa as well as for recycled concrete with density of 2334 kg/m^3^ and compressive strength of 35.5 MPa were adopted from Turk [16]. Thus, CWS and SIC-related mix proportions for 288.2 m^3^ concrete and 19.2 m^3^ concrete, respectively, were evaluated and are presented in Table 2.

### 2.3. Intensive Green Roof 

Table 3 and Table 4 detail the layers and materials required by the conventional and waste-based intensive green roofs, respectively. As was mentioned above, the roof area in both cases is 1548 m^2^. The weight of the roofs, 2340 kg_f_/m^2^ (the conventional green roof) and 1945 kg_f_/m^2^ (waste-based green roof), serves as a dead load in the seismic analysis. In addition, for both types of roofs, 490 kg_f_/m^2^ is used for live load.

In the environmental evaluations, only two different layers, substrate and drainage, were considered, omitting the other layers, as those are the same for both roof types. According to Table 3, the substrate of the conventional green roof includes gravel (2650 kg/m^3^), sand (2500 kg/m^3^), and perlite (1100 kg/m^3^). Perlite is included in the drainage layer as well. The densities of these natural materials accord those noted by SI 1045-0 [17]. According to Table 4, the substrate of the waste-based green roof includes CWAs (2385 kg/m^3^ [18]), CBA (1340 kg/m³ [19]), and FAAs (1182 kg/m^3^ [20]). FAAs are included in the drainage layer as well.

### 2.4. Seismic Response Evaluation

**Figure 4:**
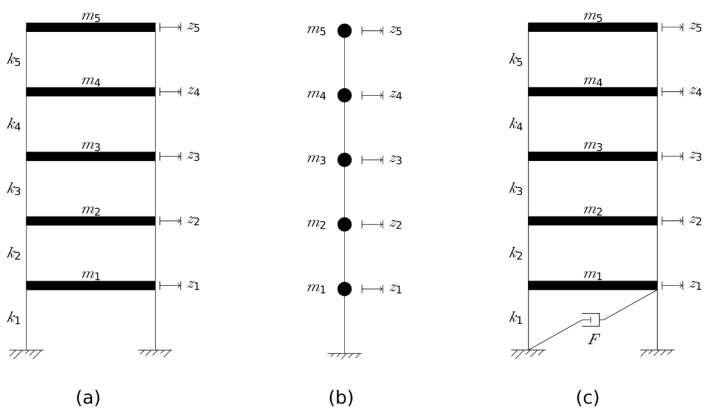
Dynamic schemes used for seismic response simulation of (**a**) the control case, (**b**) Cases 1 and 2, and (**c**) Cases 3 and 4.

Five dynamic models were formulated, one for the control case and four for Cases 1–4, taking into account each case’s special traits. Figure 4 presents dynamic schemes used for each one of the cases [12]. In this figure, z represents a dynamic-degree-of-freedom, m for the floor’s mass, k for a floor’s lateral stiffness, and F for the friction force in a friction damper. Different parameters were assigned for reflecting the dynamic attributes of each case. In the control case, the lateral stiffness of each floor is 60 times the lateral flexural stiffness of an elastic beam element, having two fixed ends and a square section made of concrete. The floor masses represent the dead load of the ceilings, flooring and infill walls, as well as 30% of the live load for residential buildings. A shear building model, given in Figure 4a, is used and its parameters are detailed in Table 5 and Table 6. Case 1’s lateral stiffness and masses are the same as in the control case, with the addition of the contribution of the shear walls and the conventional deep green roof. Assuming small displacements, the shear walls remain elastic. In the wall’s plane, their stiffness was modeled as a deep beam, connected through hinges to the original structure. The wall’s stiffness was neglected perpendicular to this plane. The stiffness of the shear walls yields a full stiffness matrix, rather than a tri-diagonal one. Hence, the model in Figure 4b is used for this case and the stiffness in its DOFs is given by the matrix:

$$K^{CWS}=\left[\begin{array}{cc} \begin{array}{cc} 6013.4 & -2221.5\\ -2221.5 & 9384 \end{array} & \begin{array}{ccc} -6.88 & 1.967 & -3.491\\ -7232.5 & -392.11 & 696 \end{array}\\ \begin{array}{cc} -6.88 & -7232.5\\ 1.967 & -392.11\\ -3.491 & 696 \end{array} & \begin{array}{ccc} 9444.3 & -1840.8 & -609.73\\ -1840.8 & 6187 & -3886\\ -609.73 & -3886 & 3678.5 \end{array} \end{array}\right],\;[\mathrm{kN}/\mathrm{mm}]$$

The mass of the retrofitting system and the conventional green roof was added to the masses of the control case. The total floor masses are detailed in Table 5. Case 2’s parameters are as in Case 1, except for the mass of the waste-based green roof that has a different density. Therefore, the same model from Case 1 is used in Case 2 with the values detailed in Table 5. In Case 3 each SIC was modeled following the approach suggested by Briman and Ribakov [14], which yielded an equivalent elastic spring coefficient and friction damper force reflecting the column properties. The total stiffness and damping of the ground floor are represented by this floor’s lateral stiffness and a friction damper with damping force *F*. The rest of the floors have stiffness similar to the control case. The same is for the model’s masses, except the fifth mass that takes into account the conventional green roof’s mass. Therefore, the model described in Figure 4c is used with the values given in Table 5 and Table 6. Case 4’s parameters are similar to those of Case 3 except for a waste-based green roof instead of the conventional one. Thus, same model from Case 3 is used for Case 4, modifying the parameters accordingly. The difference between Cases 3 and 4 in the ground floor’s horizontal stiffness emerges from the different axial forces in the SICs’ chains, leading to different lateral re-centering force. Additionally, each model was assumed to have inherent damping, typical to concrete structures. Rayleigh damping matrix was formulated for each case, assuming a damping ratio of 5% in the first two modes of vibration. The spectral traits of each model are given in Table 7. The change in the natural frequencies expresses the effect of each retrofitting. Cases 1 and 2 manifest higher frequencies compared to the control case, whereas a clear decrease is observed in Cases 3 and 4, as expected. 

These models were coded into suitable state-space models in MATLAB [21]. Whilst dynamic linear models accord the control case, Case 1, and Case 2, dynamic nonlinear models are required for Cases 3 and 4. This is due to the nonlinear behavior entailed by the friction in the SIC columns. The model’s response was computed by integrating the state equations with common LTI methods (the control case and Cases 1 and 2) or fourth order Runge-Kutta method [22] (Cases 3 and 4), for each of the given accelerograms.

As in many other works, an ensemble of past ground accelerations records was used to test the performance of each alternative [23]. It allows to examine how the inspected alternative would perform, had it been constructed and subjected to those earthquakes. This ensemble comprises Palekastro (October 2021, Greece), Nuweiba (1995, Egypt), Tabas (1978, Iran), and Erzincan (1992, Turkey) ground motions, which are accelerograms recorded in earthquakes occurred at the vicinity of discussed region. Different PGAs were recorded in these accelerograms. Hence, they were scaled to 0.29 g, for obtaining a similar comparison basis. This PGA value was set in accordance with the Israeli design code defining it as the highest design PGA in Israel [23]. The data was analyzed and transformed into common structural performance indices.

### 2.5. Environmental Evaluation

#### 2.5.1. LCA: Stages and Methodology

According to [24], a cradle-to-grave LCA of concrete components consists of: (i) design, (ii) production/execution, (iii) usage, and (iv) end of life. However, this study evaluated a cradle-to-gate LCA in the production stage only. This means that the usage and end-of-life stages were excluded from the scope of this study. The usage stage of both retrofitted alternatives was supposed to have similar lifetimes: CWS has a design lifetime of 50–70 years and SIC has a design lifetime of 50–100 years [25]. The usage stage of both roofs was also supposed to have similar maintenances during their similar lifetimes as these are related to the same extensive type of green roof. In addition, the end-of-life stage was also excluded from the evaluation because of its nonsignificant environmental damage in concrete-related cradle-to-grave LCAs [26].

Thus, goal and scope definition includes the definition of the functional unit (FU) and a description of system boundaries. An inventory analysis (life cycle inventory (LCI)) collects input and output of raw materials and emissions related to the FU, an impact assessment converts LCI results into life cycle impact assessment (LCIA) results, and an interpretation step decides on the best and worst alternatives.

#### 2.5.2. LCA: Goal and Scope

In this study, the FUs were the four retrofitted design alternatives (i.e., the materials required by Cases 1–4) that could improve the retrofitted building-related seismic performance in comparison to the seismic performance of the control case. The LCA methodology recommends that only different products/processes should be compared [27]. All the composite materials (aggregates, recycled aggregates, fly ash, cement, water, and plasticizer (Table 2)) were evaluated for the LCA analyses of Cases 1–4 of the retrofitted design alternatives. However, only the substrate and drainage layers (Table 3 and Table 4) were evaluated for the LCA analyses of Cases 1–4 of the conventional and waste-based green roofs. 

In summary, the system boundary included the production of materials for Cases 1–4 (Table 2, Table 3 and Table 4) and transportation of these materials to the building site. The transportation distance from the producer/supplier to the retrofitted building was posited as 50 km for gravel, sand, and perlite, 100 km for cement and CWAs, and 200 km for steel, plasticizer, fly ash, CBA, and FAAs.

#### 2.5.3. LCA: Life Cycle Inventory

LCIs of these retrofitting materials were based on the Ecoinvent v3.2 database and the literature sources (Table 8), and were modeled on the SimaPro platform [28]. Such secondary data were used due to the absence of local Israeli data. Nevertheless, the use of the secondary data is sufficiently appropriate for this kind of comparative study because Cases 1–4 included mostly the same building materials, such as concrete, steel, and aggregates. Note that in this study, CBA and fly ash were considered as waste from coal-fired power plants. Therefore, in concrete plant, the LCI of production processes of these wastes were omitted for Cases 1–4. LCI of FAAs production was modeled as a cold bonding process as suggested by Frankovič et al. [20]. In this process, a mixture comprising 90% FA, 10% Portland cement, and water is prepared and cured for 28 days. After that, the mixture is crushed into FAAs [20].

Table 9 shows the resulting LCI for the production of 1 kg of the materials used in Cases 1–4 and for moving these materials 1 tkm toward the building’s retrofitting site.

#### 2.5.4. LCA: Life Cycle Impact Assessment

The LCI results of Cases 1–4 were converted to LCIA results using ReCiPe 2016 [28]. ReCiPe 2016 can perform midpoint and endpoint single-score evaluations.

On the midpoint, 22 environmental impacts, including global warming (human health), global warming (terrestrial ecosystems), global warming (freshwater ecosystems), stratospheric ozone depletion, ionizing radiation, ozone formation (human health), fine particulate matter formation, ozone formation (terrestrial ecosystems), terrestrial acidification, freshwater eutrophication, marine eutrophication, terrestrial ecotoxicity, freshwater ecotoxicity, marine ecotoxicity, human carcinogenic toxicity, human non-carcinogenic toxicity, land use, mineral resource scarcity, fossil resource scarcity, water consumption (human health), water consumption (terrestrial ecosystem), and water consumption (aquatic ecosystems) can be evaluated. Among those, global warming potential, terrestrial ecotoxicity, fossil resource scarcity, and water consumption are the most significant environmental impacts for the environmental evaluation of the concretes studied here [28]. On the endpoint, the 22-midpoint evaluated environmental impacts can be grouped into damage to human health, ecosystem quality, and resources, and then converted to a single-score evaluation. The converting to single-score evaluation is performed by applying individualist (I), egalitarian (E), and hierarchist (H) perspectives on environmental problems. The I perspective evaluates only short-term damage (a 20-year time horizon), the E perspective considers all possible long-term damages (an 1000-year time horizon) and the H perspective balances between short- and long-term damage (an 100-year time horizon). In addition, the I perspective considers only substances with complete proof of their effects, the E perspective considers all reported substances, and the H perspective accounts for substances that are recognized by international health bodies. Moreover, the ReCiPe 2016 uses two types of weighting sets: a perspective-relevant (e.g., the E, H, and I weightings) and an average (A) weighting set. In the I weighting, human health is the most important damage; in the H and E weightings, ecosystem quality is the most significant damage. Taking into account all these factors, the ReCiPe 2016 converts damage to human health, ecosystem quality, and resources in individualist/average (I/A), hierarchist/average (H/A), egalitarian/average (E/A), individualist/individualist (I/I), hierarchist/hierarchist (H/H), and egalitarian/egalitarian (E/E) single-score evaluations [28]. Both midpoint and single-score evaluations have advantages and disadvantages. The midpoint evaluation has lower uncertainty, whereas the results of the endpoint single-score evaluation are much easier to interpret [29].

Consequently, the four retrofitting alternatives were evaluated using both of these methods. The midpoint H evaluation was performed considering global warming potential, terrestrial ecotoxicity, fossil resource scarcity, and water consumption. The endpoint single-score evaluation was performed applying all six evaluations: I/A, H/A, E/A, I/I, H/H, and E/E.

#### 2.5.5. Statistical Evaluation

**Figure 5:**
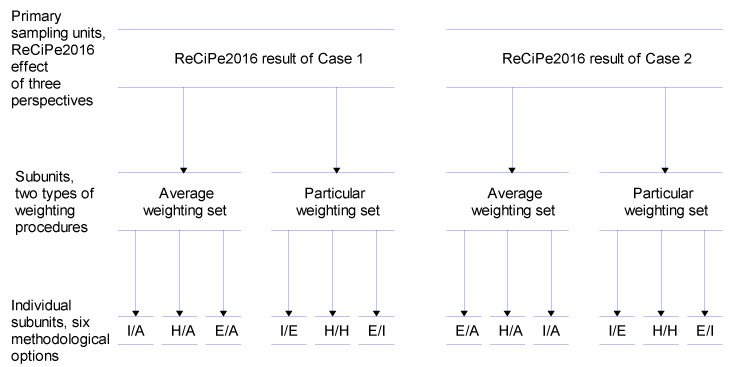
Two-stage analysis of variance (ANOVA) hierarchical design structure for environmental evaluation of Case 1 and Case 2 (ReCiPe 2016 single-score evaluation).

Figure 5 presents the application of the ReCiPe 2016 endpoint single-score design structure for comparing the LCIAs of Case 1 and Case 2. This design structure permits a pairwise comparison of the ReCiPe 2016 endpoint single-score evaluations using two-stage nested mixed ANOVA [30]. The primary sampling unit included the average and particular weighting set subunits and the two subunits utilized the six individual subunit evaluations (I/A, H/A, E/A, I/I, H/H, and E/E). This design structure was recently applied to environmental comparison between strengthened pre-stressed normal-strength concrete beams with different steel-fibered concrete layers [31].

Prior to statistical analysis, the ReCiPe 2016 endpoint single-score results were multiplied by 10^3^ and log10 transformed. Then, using the neo-Fisherian paradigm, the statistical differences between the ReCiPe 2016 endpoint single-score results of Cases 1 and 2 were compared. The neo-Fisherian paradigm (1) does not fix α, (2) does not describe *p*-values as significant or nonsignificant, (3) does not accept null hypotheses based on high *p*-values but only suspends judgment, (4) interprets significance tests according to three-valued logic, and (5) presents effect-size information if necessary [32]. As a result, the *p*-values were evaluated according to three-valued logic: either it seems to be positive (i.e., there appears to be an environmental difference between Case 1 and Case 2), it seems to be negative (i.e., there does not appear to be an environmental difference between Case 1 and Case 2), or judgment is suspended regarding the environmental difference between the two cases.

## 3. Results and Discussion

### 3.1. Seismic Response Evaluation

**Figure 6:**
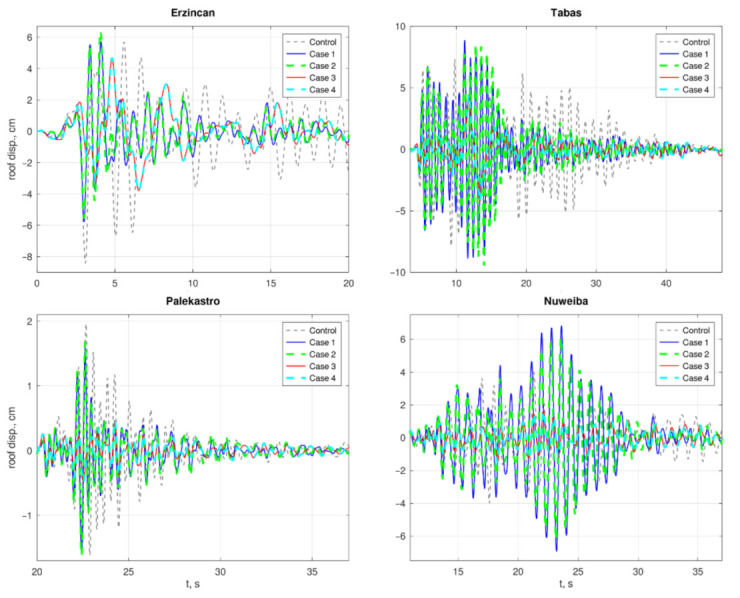
Roof displacement’s time history responses to each earthquake.

In slender buildings, roof displacements of a seismically excited structure provide a general sense of the response’s intensity. Smaller roof displacements reflect a milder response. Time histories of the roof displacements are depicted in Figure 6. As Cases 3 and 4 are base isolated, their roof displacement is increased due to the reduced rigidity of the first floor, leading to an increased displacement at the isolated floor. Hence, the roof displacements for these cases are given after offsetting the base isolation displacement, to have a better picture of the vibration intensity of the superstructure. It can be seen that Case 3 and Case 4 perform better than the others. Comparing to the control case, Cases 1 and 2 obtained an improvement of 18–31% for two earthquakes but performed worse for two others (an increase of 120–173%). Cases 3 and 4, however, performed well for all earthquakes, showing a reduction of 43–75%. 

**Figure 7:**
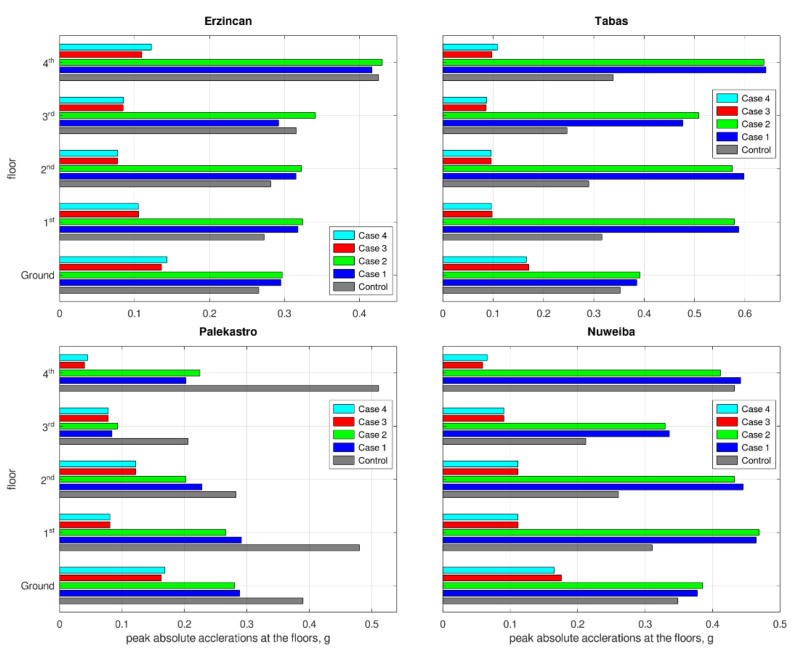
Peak floors’ acceleration responses to each earthquake.

Peak floors’ accelerations, for each earthquake, are given in Figure 7. Higher accelerations express higher loads, exerted to the structure’s floors. During earthquakes, these loads are a combination of the seismic load and the structural response, which is trying to alleviate the induced vibration. Case 3 and Case 4 experienced lower accelerations, meaning that the seismic loads are lower with such retrofittings. Thereby, Case 3 and Case 4 have responded better, in this context.

**Figure 8:**
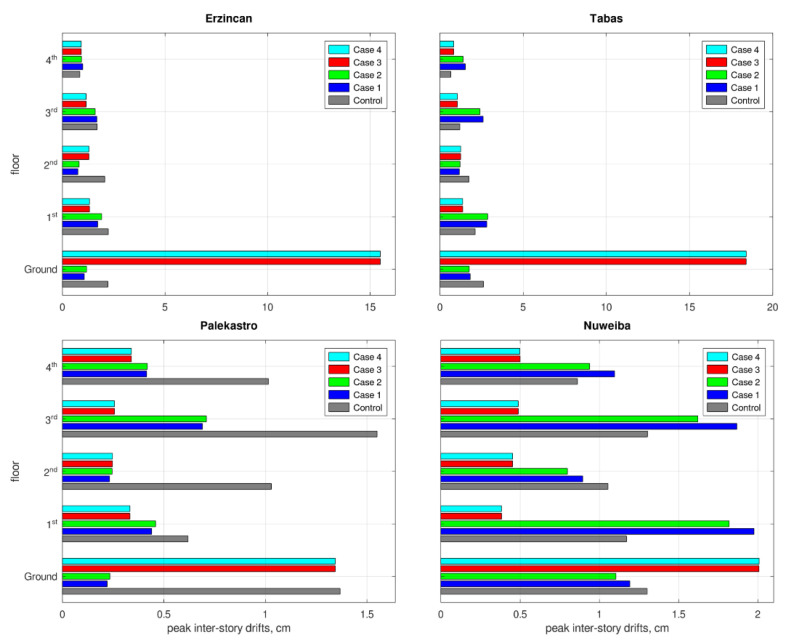
Peak inter-story drift responses to each earthquake.

**Figure 9:**
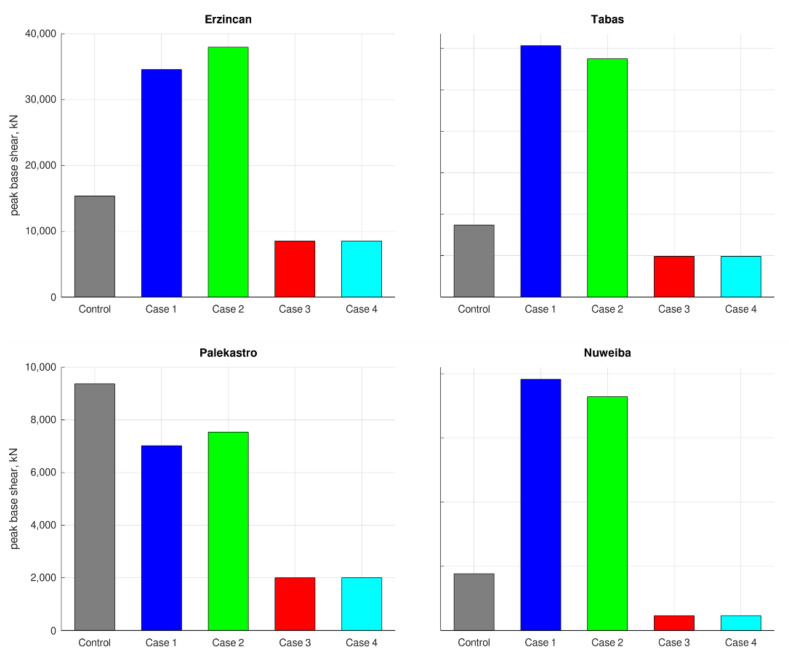
Peak base-shear responses to each earthquake.

Mechanical failure of structural elements is a major hazard during earthquakes. Intensive vibrations impose deformations on the structure’s skeleton, generate large stresses, and when these exceed the material’s limits, the structure’s stability is endangered. A prevalent approach for assessing these response components during earthquakes is to inspect the building’s inter-story drifts. Large inter-story drifts in regular structure are highly related with large bending stresses in the relevant columns. The same is true for the converse case. The peak inter-story drifts in the structure’s floors are described in Figure 8. The inter-story drifts for Case 3 and Case 4 outperformed the other cases, except for the first floor. However, this is not surprising because Case 3 and Case 4 are base isolated, allowing significant displacement to occur in the isolated floor. This phenomenon is part of the retrofitting strategy and, unlike standard floors, is not related with large stresses. Thus, the large displacements in the first floor in Case 3 and Case 4 are acceptable. Another important issue in the seismic response of structures is the loads applied to the foundations’ system. A common way to generally evaluate this is to inspect the horizontal load generated at the building’s ground floor, known as base-shear. A large base-shear means that the foundations must carry higher loads during the vibration, and if they are unable to do so, a foundation failure might follow. The peak base-shear forces are provided by Figure 9. Smaller base-shear values were observed in Cases 3 and 4, compared to the others, meaning that the foundations system is under lower risk in these cases.

Totaling the above, it can be seen that a general improvement was obtained by all the retrofitting methods, comparing to the control case. Case 3 and Case 4, however, performed much better than the others. Additionally, the responses of Case 3 and Case 4 are essentially all but identical. The same can be said for Case 1 and Case 2. This is explained by the small difference in the mechanical properties of each pair of cases, i.e., Cases 3 and 4 and Cases 1 and 2. However, there is a significant difference between the pairs due to the different retrofitting approaches.

### 3.2. Environmental Evaluation

#### 3.2.1. ReCiPe 2016 Midpoint Results

**Figure 10:**
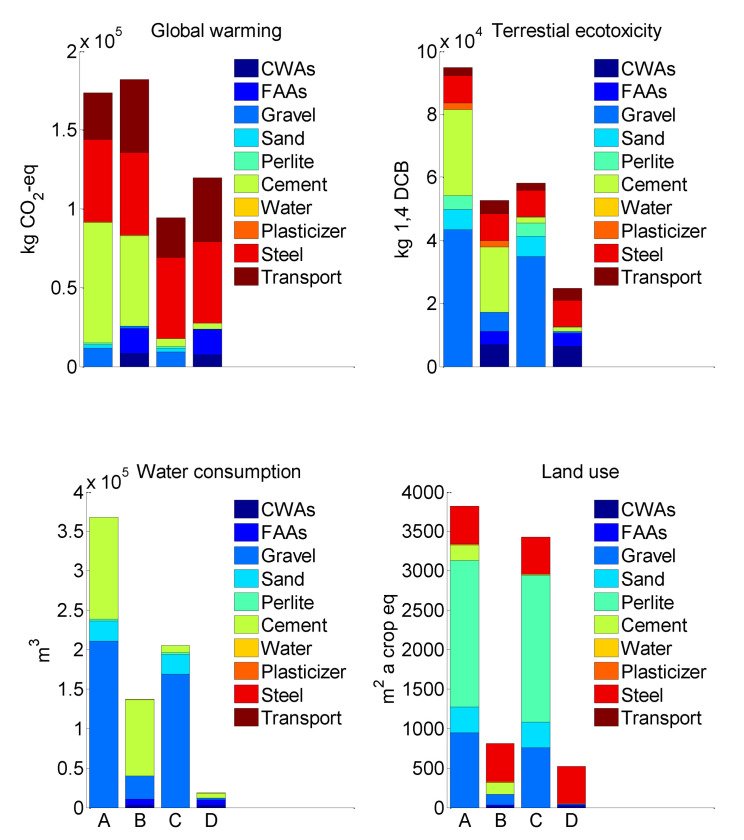
(**A**) Case 1: concrete wall strengthening (CWS)-conventional concrete + conventional green roof, (**B**) Case 2: CWS-waste-included concrete + waste-based green roof, (**C**) Case 3: seismic isolation columns (SIC)-conventional concrete + conventional green roof, and (**D**) Case 4: SIC-waste-included concrete + waste-based green roof evaluated with ReCiPe 2016 midpoint method.

Figure 10 shows that, comparing to Cases 1 and 3 (CWS and SIC conventional concrete-based retrofitting methods, respectively, with conventional green roofs), Case 2 and Case 4 (CWS and SIC waste-based retrofitting methods, respectively, with waste-based roofs) have lower terrestrial ecotoxicity, water consumption, and land use. On the other hand, Case 1 and Case 3 have lower global warming potential. In particular, compared to Case 1, the impact of ecotoxicity, water consumption, and land use of Case 2 are lower by 44, 63, and 79%, respectively, and compared to Case 2, the impact of the global warming potential of Case 1 is lower by 5%. Similarly, compared to Case 3, the impact of ecotoxicity, water consumption, and land use of Case 4 is lower by 58, 91, and 85%, respectively, and compared to Case 4, the impact of the global warming potential of Case 3 is lower by 21%.

These differences in impacts are due to different materials, natural and waste-based, used for the CWS and SIC retrofitting methods as well as for the green roofs (Table 2, Table 3 and Table 4). The natural material-based retrofitting methods with natural material-based green roofs (Case 1 and Case 3) mostly rely on the use of conventional concrete, steel, and natural aggregates (gravel, sand, and perlite), whereas the waste-based retrofitting methods with waste-based green roofs (Case 2 and Case 4) mostly rely on the use of steel and involve additional transport to deliver the wastes to the building site.

Among three basic concrete components, such as aggregates, water, and cement, the cement production process is the largest consumer of energy and producer of the highest amount of CO_2_ emissions [33]. To produce clinker, calcareous and argillaceous materials need to be heated to 1450 °C. If the heating energy is produced with burning fossil fuels (oil, coal, or gas), a large amount of CO_2_ is released into the atmosphere. As well, the clinker-related calcination process itself is responsible for an additional release of CO_2_ into the atmosphere [34]. The high CO_2_ emissions of cement production was recognized by many research studies, which confirmed that 74–93% of the total CO_2_ is released from concrete production [35,36,37]. Cement production also requires using a large amount of water during grinding and mixing raw calcareous and argillaceous materials for clinker production. In this way, cement production is a huge contributor to GWP, TE, and WC impacts (Figure 10, Case 1 and Case 2 are based on large amounts of concrete).

Steel production is another noticeable contributor to GWP, TE, and LU (Figure 10, Cases 1–4). This is because almost the same steel quantity was used in both retrofitting methods, 22.5 tons in CWS and 22.3 tons in SC, in combination with conventional green roofs and waste-based green roofs. As was noted by Liang et al. [38], the steel-making blast furnace process, which is responsible for 70% of steel produced all over the world, mostly contributes to the global warming potential (40%), abiotic depletion potential (35%), and human toxicity potential (17%). In addition, according to the LCI results, the production of 1 kg of steel from iron ore mined from Earth required 0.0211 m^2^ of crop equivalent land use, thereby resulting in high LU impact for all four retrofitted cases (Table 4).

Natural aggregates (gravel, sand, and perlite) that were mostly used for Cases 1 and 3 contribute to TE, WC, and LU (Figure 10). In contrast, GWP, which is related to the production of natural aggregates, was found to be smaller by 9% and 13% of the total concrete related GWP for CWS and SIC methods, respectively. These results are in line with the results presented by other research studies, which confirmed that aggregate production is responsible for 2 to 20% [37] of the total concrete’s CO_2_ emissions. 

Wastes used in FA, which was used in waste-based concrete of retrofitting methods, and CBA, which was used in waste-based green roofs, are considered here as wastes from coal-burning electricity production process. Therefore, only the impacts related to the transportation of these wastes to the building site were accounted. As a result, the GWP pertaining transporting in Case 2 and Case 4 is higher than the GWP related to transport of Case 1 and Case 3. 

#### 3.2.2. ReCiPe 2016 Endpoint Single-Score Results

**Figure 11:**
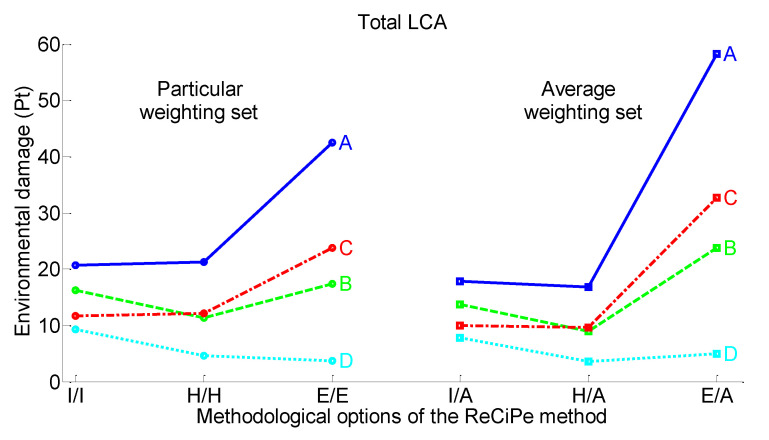
(**A**) Case 1: concrete wall strengthening (CWS)-conventional concrete + conventional green roof, (**B**) Case 2: CWS-waste-included concrete + waste-based green roof, (**C**) Case 3: seismic isolation columns (SIC)-conventional concrete + conventional green roof, and (**D**) Case 4: SIC-waste-included concrete + waste-based green roof evaluated with ReCiPe 2016 endpoint single-score method.

Figure 11 shows that Case 4 is the best retrofitted alternative with the smallest environmental damage, while Case 1 is the worst retrofitted alternative with the biggest environmental damage. Moreover, the difference between these two cases holds for all six methodological options (I/A, H/A, E/A, I/I, H/H, and E/E). However, the results for Case 2 and Case 3 are varying with respect to the different methodological options. Case 2 evaluated with E/E and E/A options has lower environmental damage than Case 3, whereas evaluation with I/I and I/A options reveals that Case 3’s environmental damage is lower than that of Case 2. 

Table 10 demonstrates *p*-values of the differences in single-score evaluation (simultaneous evaluation of the six methodological options) between pairs of four retrofitted design alternatives. According to these results, a negative difference exists only when comparing Case 2 against Case 3 (*p* = 0.4141), whereas there are positive differences between the other compared pairs, such as Case 1 and Case 2, Case 1 and Case 3, Case 1 and Case 4, Case 2 and Case 4, and Case 3 and Case 4 (0.0002 ≤ *p* ≤ 0.0011).

This means that according to evaluation conducted by the ReCiPe 2016 endpoint single-score method, Case 4 is the first preferable alternative as a less damaging retrofitting method. This conclusion is independent of the applied perspective with regard to different views on the significance of the environmental problem. The next preferable alternatives are Case 2 and Case 3 as no environmental difference was revealed between this pair of the retrofitting methods. Finally, all six methodological options point out Case 1 as the most damaging retrofitting method, hence, the most inappropriate alternative.

## 4. Conclusions

This study evaluated seismic and environmental performances of four retrofitting methods: Case 1: concrete wall strengthening (CWS)-conventional concrete + conventional green roof, Case 2: CWS-waste-included concrete + waste-based green roof, Case 3: seismic isolation columns (SIC)-conventional concrete + conventional green roof, and Case 4: SIC-waste-included concrete + waste-based green roof.

According to the seismic analysis, Case 1 and Case 2 demonstrated certain small improvements in the seismic-bearing capacity of the retrofitted building. Thus, incorporation of an intensive green roof, as an environmental enhancement that is incorporated into the seismic design of such typical buildings in Israel, can be considered as a positive measure. 

However, much better improvements in the seismic-bearing capacity of the retrofitted building was shown in Cases 3 and 4. Thus, according to the seismic analysis, using SIC is favorable for the seismic retrofitting of typical old Israeli buildings with the described attributes. Moreover, that fact is not diminished by the existence of an intensive green roof in the building.

According to the environmental performance results evaluated with the hierarchist (H) methodological option of the ReCiPe 2016 midpoint, Case 2 and Case 4 (CWS and SIC waste-based retrofitting methods, respectively, with waste-based roofs) had lower terrestrial ecotoxicity, water consumption, and land use, compared to Case 1 and Case 3 (CWS and SIC conventional concrete-based retrofitting methods, respectively, with conventional green roofs). Case 1 and Case 3, however, had lower global warming potential. Thus, replacing conventional concrete with waste-based concrete in CWS and SIC retrofitting methods, and natural aggregates with recycled aggregates in intensive green roofs, can improve three of the four environmental impacts considered in this study.

Furthermore, the ReCiPe 2016 endpoint single-score results allowed to compare Case 2 against Case 4 and pointed out Case 4 as the best retrofitting alternative. For this evaluation, the environmental preferability of Case 4 was confirmed for all six methodological options (I/A, H/A, E/A, I/I, H/H, and E/E). It was found that the environmental damage caused by production processes related to Case 4 was significantly lower than that of Case 2. This result was found to be consistent across three interpretations (egalitarian, individualist, and hierarchist) of the significance of the environmental problem. The next preferable alternatives were Case 2 and Case 3. It is interesting that comparing to Case 2 (in which CWS was built with waste-included concrete and waste-based green roof), Case 3 (in which SIC was built with conventional concrete and green roof with natural aggregates) had similar environmental damage. This is because of the lower concrete quantity used by the SIC method. However, as was already noted, the seismic performance of Case 3 was much better compared to that of Case 2. 

Thus, according to both seismic and environmental performances, Case 4 retrofitting alternative, which includes SIC retrofitting, waste-included concrete, and an intensive green roof built with recycled aggregates, can be classified as the best building retrofitting method. These findings were obtained for a class of buildings located in Israel and may not be applicable to buildings with different configurations in other locations. However, it demonstrates that as the construction industry uses many other building-related retrofitting measures, seeking to improve the seismic durability and the environmental performance of the applied measures should not be ignored.

## Figures and Tables

**Table 1:** Case studies.

	Retrofitting Method	CWS	SIC
Materials			
Conventional concrete + conventional intensive green roof		Case 1	Case 3
Waste-included concrete + waste-based intensive green roof		Case 2	Case 4

**Table 2:** Materials required for retrofitting with CWS and SIC-based conventional and waste-included concretes.

Retrofitting Method	Aggregates (kg)	Recycled Aggregates (kg)	Fly Ash (kg)	Cement (kg)	Water (kg)	Plasticizer (kg)
CWS-conventional concrete	555,073	–	–	92,224	50,435	576
CWS-waste-included concrete	365,726	156,781	23,056	69,168	57,352	576
SIC-conventional concrete	36,979	–	–	6144	3360	38
SIC-waste-included concrete	24,365	10,445	1536	4608	3821	38

**Table 3:** Materials required for the conventional green roof.

Layer	Thickness (m)	Material	Weight (kg/m^2^)	Weight (kg/Roof Area)
*Substrate*	1	*Gravel*	1325	*2,051,100*
		*Sand*	625	*967,500*
		*Perlite*	275	*425,700*
Filter layer	–	Polypropylene sheet	0.60	–
*Drainage*	0.1	*Perlite*	110	*170,280*
Protection layer	–	Polypropylene sheet	0.60	–
Roof barrier	0.0004	HDPE	0.385	–
Insulation	0.05	Polystyrene	1.5	–
Total	–	–	2340	–

Note: HDPE–high-density polyethylene. Italic font—layers with their respective weights that were included in the LCA evaluations.

**Table 4:** Materials required for the waste-based green roof.

Layer	Thickness (m)	Material	Weight (kg/m^2^)	Weight (kg/Roof Area)
Substrate	1	*CWAs*	1193	*1,846,764*
		*CBA*	335	*518,580*
		*FAAs*	296	*458,208*
Filter layer	–	Polypropylene sheet	0.60	–
Drainage	0.1	*FAAs*	118	*182,664*
Protection layer	–	Polypropylene sheet	0.60	–
Roof barrier	0.0004	HDPE	0.385	–
Insulation	0.05	Polystyrene	1.5	–
Total	–	–	1945	–

Note: CWAs–concrete waste-based aggregates, CBA–coal bottom ash, FAAs–fly ash-based aggregates, HDPE–high-density polyethylene. Italic font—layers with their respective weights that were included in the LCA evaluations.

**Table 5:** Mass values used in the dynamic models of each of the cases.

	Control Case	Case 1	Case 2	Case 3	Case 4
$m_{1}$, ton	1904	2066	2066	1904	1904
$m_{2}$, ton	1904	2066	2066	1904	1904
$m_{3}$, ton	1904	2036	2036	1904	1904
$m_{4}$, ton	1904	2036	2036	1904	1904
$m_{5}$, ton	1333	5846	5234	5714	5102

**Table 6:** Stiffness and friction values used in the dynamic models of the control case, Case 3 and Case 4.

	Control Case	Case 3	Case 4
$k_{1}$, kN/mm	658.1	46.245	46.245
$k_{2}$, kN/mm	658.1	658.1	658.1
$k_{3}$, kN/mm	658.1	658.1	658.1
$k_{4}$, kN/mm	658.1	658.1	658.1
$k_{5}$, kN/mm	658.1	658.1	658.1
F, kN	–	1255.2	1198

**Table 7:** Natural frequencies and mode shapes for each case.

	$f_{n},\;\mathrm{Hz}$	$\varphi^{T}$				
Control case	0.89	0.17	0.33	0.46	0.55	0.59
	2.6	0.46	0.57	0.24	−0.27	−0.58
	4	0.58	0.083	−0.57	−0.16	0.55
	5.1	0.51	−0.49	−0.026	0.52	−0.48
	5.7	0.28	−0.49	0.57	−0.51	0.31
Case 1	1.2	0.12	0.31	0.39	0.56	0.65
	4.3	0.33	0.67	0.6	0.063	−0.28
	8.7	0.89	−0.041	−0.26	−0.36	0.11
	9.7	0.34	−0.26	−0.23	0.86	−0.19
	14	0.14	−0.69	0.7	−0.091	−0.012
Case 2	1.2	0.12	0.31	0.39	0.56	0.65
	4.3	0.33	0.67	0.59	0.039	−0.3
	8.7	0.89	−0.051	−0.27	−0.34	0.12
	9.8	0.31	−0.25	−0.22	0.86	−0.21
	14	0.14	−0.69	0.7	−0.09	−0.014
Case 3	0.28	0.4	0.43	0.45	0.47	0.48
	1.5	0.68	0.54	0.27	−0.082	−0.41
	3.2	0.58	−0.042	−0.62	−0.49	0.2
	4.6	0.43	−0.58	−0.18	0.66	−0.1
	5.6	0.23	−0.57	0.65	−0.45	0.046
Case 4	0.28	0.41	0.43	0.45	0.47	0.48
	1.6	0.67	0.53	0.24	−0.11	−0.44
	3.2	0.58	−0.052	−0.62	−0.47	0.22
	4.6	0.43	−0.58	−0.17	0.66	−0.12
	5.6	0.23	−0.56	0.65	−0.45	0.053

Note: The mode shapes are written as row vectors, next to the pertaining natural frequency.

**Table 8:** Production stage: data input (Ecoinvent v3.2, SimaPro v9.1, 2019) for modeling life-cycle inventory (LCI) for Cases 1–4.

Material/Process	Process
Gravel	Gravel, crushed, at mine/CH U
Sand	Sand, at mine/CH U
Perlite	Perlite, at mine/DE U
Cement	Portland cement, strength class Z 42.5, at plant/CH U
CWAs	Disposal, building, concrete, not reinforced, to recycling/CH U
Water	Top water, at user/CH U
FAAs	FAAs, production (modeled according to Frankovič et al. [17]).
Plasticizer	Polycarboxylates, 40% active substance (RER)
Steel	Steel rebar/EU
Transportation	Lorry transport, Euro 0, 1, 2, 3, 4 mix, 22 t total weight, 17.3 t

Note: CWAs, concrete waste-based aggregates; FAAs, fly ash-based aggregates. FAAs were modeled on SimaPro platform according to Frankovič et al. [20].

**Table 9:** Life-cycle inventory (LCI) for assessment of Cases 1–4 (Ecoinvent v3.2 database).

Material/Process	GWP (kg CO_2_)	TE (kg 1,4-DCB)	WC (m^3^)	LU (m^2^a crop eq)
Gravel	0.00445	0.0167	0.081	0.000362
Sand	0.00242	0.0067	0.026	0.000337
Perlite	0.00169	0.00706	0.00409	0.00312
Cement	0.828	0.297	1.4	0.00218
Water	0.000171	0.000409	0.00448	0.0000166
CWAs	0.00403	0.00343	0.00136	0.00000676
FAAs	0.025	0.00661	0.0113	0.000031
Plasticizer	1.15	3.75	0.0137	0.0204
Steel	2.31	0.381	0.00246	0.0211
Transportation	0.0663	0.00587	0.00000529	0

Note: GWP, global warming potential; FRS, fossil resource scarcity; WC, water consumption; TE, terrestrial ecotoxicity; 1,4-DCB, 1,4-dichloro-benzine equivalent.

**Table 10:** *p*-values of the differences in single-score evaluation between pairs of four retrofitted design alternatives for the life-cycle assessment (LCA) (production stage). The LCAs were evaluated via the six ReCiPe2016 single-score methodological options.

Retrofitted Design Alternatives	Case 1	Case 2	Case 3	Case 4
Case1: CWS-conventional concrete + conventional green roof	X	**0.0011**	**0.0010**	**0.0002**
Case 2: CWS-waste-included concrete + waste-based green roof		X	0.4141	**0.0006**
Case 3: SIC-conventional concrete + conventional green roof			X	**0.0005**
Case 4: SIC-waste-included concrete + waste-based green roof				X

Note: Normal font represents a negative difference between the compared cases; bold font represents a positive difference between the compared cases.
